# Diuretic Treatment in Heart Failure: A Practical Guide for Clinicians

**DOI:** 10.3390/jcm13154470

**Published:** 2024-07-30

**Authors:** Lingling Wu, Mario Rodriguez, Karim El Hachem, Chayakrit Krittanawong

**Affiliations:** 1 Cardiovascular Division, University of Alabama at Birmingham, Birmingham, AL 35294, USA; 2 John T. Milliken Department of Medicine, Division of Cardiovascular Disease, Section of Advanced Heart Failure and Transplant, Barnes-Jewish Hospital, Washington University in St. Louis School of Medicine, St. Louis, MO 63110, USA; 3 Division of Nephrology, Icahn School of Medicine at Mount Sinai, Mount Sinai Hospital, New York, NY 10029, USA; 4 Section of Cardiology, Cardiology Division, NYU Langone Health and NYU School of Medicine, 550 First Avenue, New York, NY 10016, USA

**Keywords:** diuretic, heart failure

## Abstract

Congestion and fluid retention are the hallmarks of decompensated heart failure and the major reason for the hospitalization of patients with heart failure. Diuretics have been used in heart failure for decades, and they remain the backbone of the contemporary management of heart failure. Loop diuretics is the preferred diuretic, and it has been given a class I recommendation by clinical guidelines for the relief of congestion symptoms. Although loop diuretics have been used virtually among all patients with acute decompensated heart failure, there is still very limited clinical evidence to guide the optimized diuretics use. This is a sharp contrast to the rapidly growing evidence of the rest of the guideline-directed medical therapy of heart failure and calls for further studies. The loop diuretics possess a unique pharmacology and pharmacokinetics that lay the ground for different strategies to increase diuretic efficiency. However, many of these approaches have not been evaluated in randomized clinical trials. In recent years, a stepped and protocolized diuretics dosing has been suggested to have superior benefits over an individual clinician-based strategy. Diuretic resistance has been a major challenge to decongestion therapy for patients with heart failure and is associated with a poor clinical prognosis. Recently, therapy options have emerged to help overcome diuretic resistance to loop diuretics and have been evaluated in randomized clinical trials. In this review, we aim to provide a comprehensive review of the pharmacology and clinical use of loop diuretics in the context of heart failure, with attention to its side effects, and adjuncts, as well as the challenges and future direction.

## 1. Introduction

Congestion and fluid retention are the hallmarks of decompensated heart failure (HF) and the major reason for the hospitalization of patients with heart failure [1]. Diuretics have been used in heart failure for decades, and they remain the backbone of the contemporary management of heart failure. Loop diuretics is the preferred diuretic, and it has been given a class I recommendation by clinical guidelines for the relief of congestion symptoms [2,3]. Although loop diuretics have been used virtually among all patients with acute decompensated heart failure, there is still very limited clinical evidence to guide the optimized diuretics use [4]. This is a sharp contrast to the rapidly growing evidence of the rest of the guideline-directed medical therapy of heart failure and calls for further studies. The loop diuretics possess a unique pharmacology and pharmacokinetics that lay the ground for different strategies to increase diuretic efficiency. However, many of these approaches have not been evaluated in randomized clinical trials (RCTs). In recent years, a stepped and protocolized diuretics dosing has been suggested to have superior benefits over an individual clinician-based strategy [5]. Diuretic resistance has been a major challenge to decongestion therapy for patients with heart failure and is associated with poor clinical prognosis [6]. Recently, therapy options have emerged to help overcome diuretic resistance to loop diuretics and have been evaluated in randomized clinical trials. In this narrative review, we aim to provide a comprehensive review of the pharmacology and clinical use of loop diuretics in the context of heart failure, with attention to its side effects, and adjuncts, as well as the challenges and future direction. A literature review was conducted through a search of MEDLINE and EMBASE databases using the following keywords: “heart failure; diuretics; clinical trial; review; meta-analysis; guideline” until 30 June 2024. Excluded were studies with only abstracts available or studies that focused on diuretics use among patients with hypertension only. 

## 2. Loop Diuretics—Pharmacology

The kidney plays a vital role in maintaining the fluid and electrolyte balance in the body. Heart failure alternates the physiological process of renal filtration and reabsorption and triggers the maladaptation of the kidney through the activation of the neurohormone system [7]. These neurohormones often enhance the reabsorption of sodium and water to increase the preload of the left ventricle but also increase fluid retention and contribute to congestion symptoms [7]. Loop diuretics are the most potent diuretics and are the preferred agent in congestive heart failure [2,3]. Loop diuretics reversibly and competitively inhibit sodium-potassium-chloride cotransporter-2 (NKCC2) on the luminal side of the thick ascending loops’ epithelial cell, which accounts for 25–30% of the reabsorption of filtered NaCl from glomerular membranes [8]. As a result, the reabsorption of sodium and chloride declines, and tubular fluid becomes more hypertonic, which diminishes the osmotic gradient required for water reabsorption [8]. Loop diuretics also act on the NKCC1 cotransporter, which is expressed in vascular smooth muscle cells, and explain its vasodilatory effect [9]. Lastly, loop diuretics uniquely act on the distal part of the loop of Henle, which contains macula densa where the NKCC2 co-transporter is also expressed [10]. The inhibition of the NKCC2 co-transporter results in decreased sodium sensing from macula densa and promotes renin–angiotensin–aldosterone activation and sodium/water reabsorption in the proximal and distal tubule [11]. This compensatory hormonal response acts as a natural counterbalance to loop diuretics and contributes to diuretic resistance [12]. When loop diuretics are given to a patient who is naïve to diuretics, the hormonal response will not be sufficient compared to the overwhelmingly increased delivery of sodium to the distal tubule. Thus, the diuretic response is often robust. In comparison, distal tubule hypertrophy is frequently observed among patients on chronic loop diuretics. Therefore, the diuretic response is more blunted in such patients [13]. 

## 3. Loop Diuretic Pharmacokinetics

Different loop diuretics possess different pharmacological properties and pharmacokinetics. When given orally, loop diuretics are absorbed from the gastrointestinal tract and have various amounts of bioavailability depending on the agent. Furosemide has an unpredictable absorption and, on average, a lower bioavailability of 50%, whereas torsemide and bumetanide have a more predictable absorption and a high bioavailability (>80%) [14]. In the face of severe venous congestion and gut edema during acute decompensated heart failure, the intestinal absorption for loop diuretic can be further slowed, hence often requiring intravenous administration [15]. The exception is torsemide, which typically does not come in intravenous form and is better absorbed from the gastrointestinal tract compared to the rest of the loop diuretics [16]. When administrated intravenously, loop diuretics have a much faster onset, typically within 10 min versus 30 min to 1 h when taken orally [17]. Loop diuretics are highly bonded by albumin and will be transported to the kidney via blood flow, where it gains access to the tubular fluid by active proximal secretion via organic anion transporters (OATs) on the basolateral membrane and multidrug resistance-associated protein 4 (MRP4) on the luminal membrane of the proximal tubule [18]. Defects in tubular function, increased plasma organic anions such as the uremic anion, concomitant non-steroidal anti-inflammatory drug (NSAID) use, or hypoalbuminemia can negatively affect the active secretion process and delivery of the loop diuretics to thick ascending limb tubules [19]. The half-life of furosemide is about 1.5–2 h, whereas the half-life of bumetanide is shorter at 1 h, and that of torsemide is longer at 3–4 h [20]. Due to their shorter half-life, furosemide and bumetanide typically require at least a twice-a-day dosing schedule, whereas torsemide can be given once daily. The metabolism process also differs between loop diuretics. Torsemide and bumetanide are primarily metabolized through the liver, with lesser amounts through the kidney. In contrast, furosemide is primarily metabolized through glucuronidation in the kidney and, therefore, has a longer half-life in kidney failure [17]. 

Loop diuretics have a sigmoid-shaped dose–response curve which means the loop diuretics have a very minimal effect before reaching the threshold dose, beyond which point the loop diuretics rapidly approach the maximum effect with a dose increase, often termed the “ceiling effect” [19,21]. However, an increased diuretics dose above the ceiling dose will still produce more natriuresis because it will allow serum concentrations to be higher above the threshold dose for a longer time [20]. The pharmacokinetics features of loop diuretics underscore the recommendation of doubling the dose of the diuretic when resistance is met, as the dose–response curve is not linear but logarithmic. The dose–response curve is often shifted rightwards and downwards in acute decompensated heart failure, which increases the threshold dose and lowers the ceiling dose due to the attenuated natriuretic response to loop diuretics [22]. The continuous infusion of loop diuretics is theorized to have a better diuretic response and fewer side effects due to more stable serum concentrations above the threshold for a longer period compared to bolus administration. However, this has not been proven in the prospective clinical study [23]. The individual response to a fixed dose of diuretics varies and is often impacted by many factors contributing to diuretic resistance (discussed later). To describe the clinical response of heart failure patients to diuretics doses, the term “diuretics efficiency”, which is typically defined as net fluid loss per 40 mg of furosemide, has been used in recent years in clinical trials as an important clinical outcome in evaluating different diuretic strategies [24,25]. The variability of the threshold dose and response to diuretics among patients with heart failure also calls for a stepwise diuretic strategy, which will be discussed below. 

## 4. Loop Diuretic Use in Acute Decompensated Heart Failure

Congestion plays a central role in the pathogenesis of decompensated heart failure and contributes to the majority of the symptoms and hospitalization from heart failure [26]. Loop diuretics are the cornerstone of decongestion and have been recommended by clinical guidelines as the first-line therapy in congestive heart failure [2,3]. Despite its wide use, the clinical evidence of how to achieve decongestion effectively and safely remained limited. The DOSE trial evaluated intermittent vs. continuous IV loop diuretics and low-dose (IV equivalent of oral home dose) vs. high-dose (2.5× home dose) loop diuretics among patients with acute decompensated heart failure and found that, although there was no difference in the primary outcome (global assessment of symptoms and change in creatinine) at 72 h, it did show that high-dose loop diuretics was associated with a greater relief of dyspnea, weight loss, and net fluid loss [23] (Table 1). In the CARRESS-HF trial, a stepped pharmacological approach (urine-output-based) of diuretics was compared against ultrafiltration in acute decompensated heart failure. The stepped pharmacologic therapy algorithm of diuretics was found to be superior to ultrafiltration (UF) for the preservation of renal function at 96 h, with a similar amount of weight loss [5]. A post hoc analysis of the CARRESS-HF trial (stepped and protocolized approach) against the DOSE and ROSE-AHF trial (non-protocolized and clinicians-directed approach) revealed that a stepped diuretics dose strategy is associated with a greater net fluid and weight loss without being associated with renal compromise [27]. The urine output or net-fluid-based algorithm of diuretics dosing can be challenging due to inaccuracies and delays in data collection, thus limiting the ability to detect non-responders early. A natriuresis-based assessment has recently been suggested to overcome the limitation of the net-fluid-based assessment of the diuretic response [28]. Spot urine (first voided urine) sodium of less than 50–65 mEq/L was found to be associated with an inadequate diuretic response and adverse clinical outcomes [29,30]. A recent trial using protocolized natriuresis-guided decongestion (ENACT-HF trial) has been shown to be associated with a better urine output and shorter length of stay among patients with heart failure [31].

**Figure 1:**
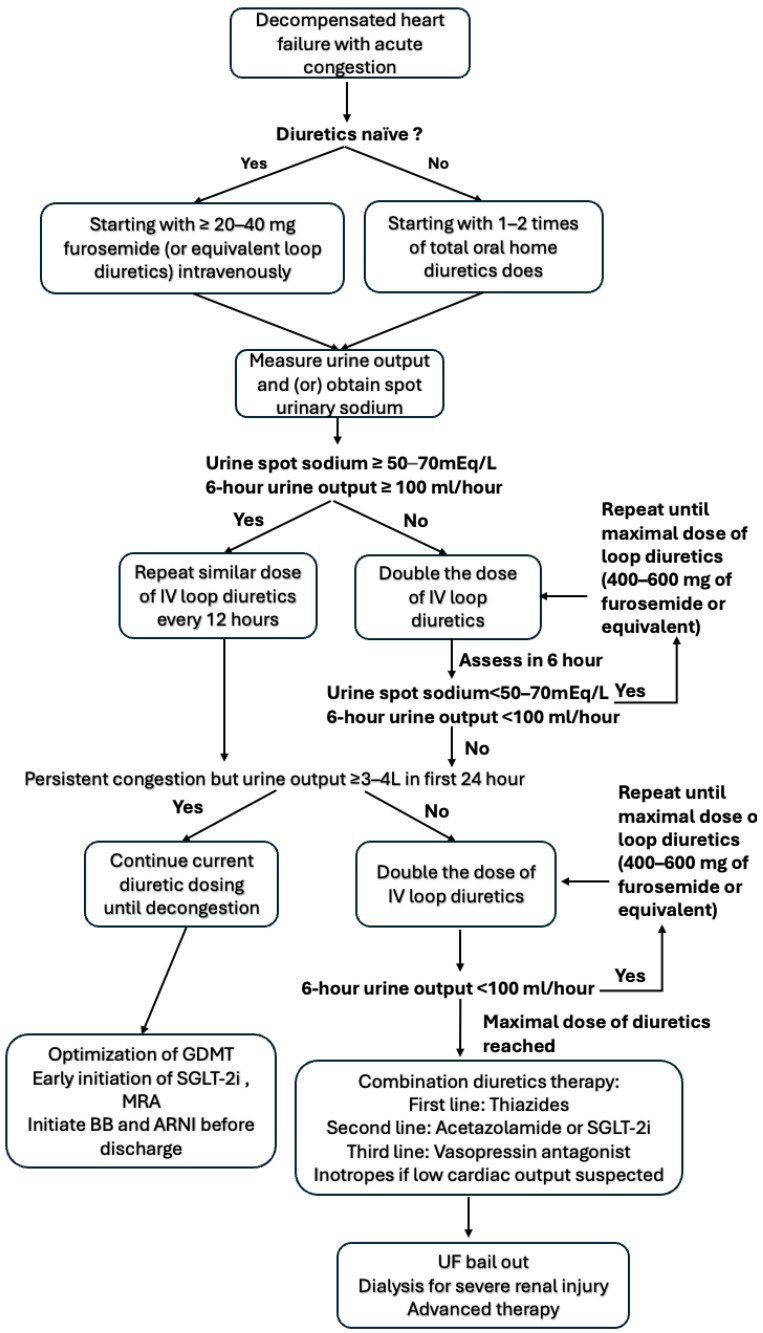
Algorithm stepwise approaches for how to use diuretics in ADHF (adapted from 2021 ESC HF guideline).

Based on emerging clinical evidence, a stepwise approach for the titration of diuretics in decompensated heart failure using a urine output and natriuresis-based approach has been proposed by ESC (Figure 1) [2]. Loop diuretics should be administered intravenously, and the starting dose for diuretics-naïve patients should be at least 20–40 mg and 1–2 times the total oral home dose diuretics for those already on home diuretics. After the first dose of diuretics, urine should be collected, and the total output should be measured. If the spot urine sodium is less than 50–70 mEq/L or urine output is less than 100 mL/h at 6 h, the loop diuretics dose should be doubled until the appropriate response or maximum dose (total daily dose of 400–600 mg furosemide or equivalent dose of loop diuretics) is reached, at which point an every 8 to 12 h dosing schedule can be applied and continued until decongestion. For those with persistent congestion with a urine output of less than 3–4 L in 24 h with a high dose or maximized loop diuretics dosing, further adjuncts of loop diuretics such as thiazide (first line), acetazolamide, or the sodium-glucose transport protein 2 (SGLT-2) inhibitor should be utilized. Ultrafiltration (UF) and advanced heart failure therapy should also be considered if the above strategy fails, and are generally associated with worse clinical outcome [6]. Loop diuretics administration can cause neurohormone activation, which is detrimental to acute heart failure [32]. Therefore, diuretics should not be given alone without the continuation/early initiation of other neurohormone modulators, such as Angiotensin receptor neprilysin inhibitor (ARNI), mineralocorticoid receptor antagonists (MRAs), beta-blockers (BBs), and SGLT-2-inhibitors.

Once euvolemia is reached, an assessment should be made for the long-term diuretic needs, and a transition to oral diuretics should be planned. Clinical guidelines recommend transitioning to an oral diuretics dose at the lowest dose possible to avoid congestion [2]. There is no consensus about the dosing strategy for transitioning from intravenous to oral diuretics. For those already on home diuretics, a retrospective study has shown that an increase in the loop diuretic dose from the prehospitalization dose at discharge was associated with fewer 30-day readmissions [33]. It has also been suggested by guidelines that patients should be observed in the hospital for 24 h on oral diuretics before discharge [2]. However, this has been challenged by recent studies that failed to show any significant benefit with this approach [34,35].

## 5. Loop Diuretic Use in Chronic Congestive Heart Failure

Diuretics are recommended by clinical guidelines for chronic congestive heart failure patients to prevent symptoms from congestion [2,3]. Furosemide is the most used agent due to its vast clinical experience and availability [36]. Some clinicians favor torsemide and bumetanide due to their better bioavailability and/(or) longer half-life. Early studies have suggested that torsemide might be superior to furosemide [37,38]. This was investigated in a recent Transform HF trial, which failed to demonstrate the benefit of torsemide over furosemide for all-cause mortality over 12 months [39]. Current clinical guidelines recommend using the lowest possible maintenance diuretic dose for chronic heart failure patients, as diuretics can be associated with side effects, including volume depletion and electrolyte derangement [3]. The dose of diuretics often changes over time, as it is shown in the CHAMPION trial [40]. Therefore, it is important to monitor and schedule regular follow-up visits after the discharge from acute hospitalization. An improved hemodynamical profile and lowered dose requirement of diuretics was observed frequently in heart failure patients after the initiation of guideline-directed medical therapy [41]. An observational study suggests chronic congestive heart failure patients without diuretics requirement have a better prognosis than those who require diuretics [42]. The chronic use of diuretics can also cause side effects such as electrolyte derangement and hypovolemia. Therefore, the active down-titration of diuretics has been suggested to be beneficial for chronic heart failure patients. A recent trial that enrolled 417 stable heart failure patients on low-dose diuretics showed that the withdrawal of diuretics in the low-risk heart failure population was not associated with worsening heart failure symptoms or increased heart failure hospitalizations [43]. The down-titration of loop diuretics at the time of heart failure discharge has also been shown to have no association with increased rehospitalization or mortality [44].

**Figure 2:**
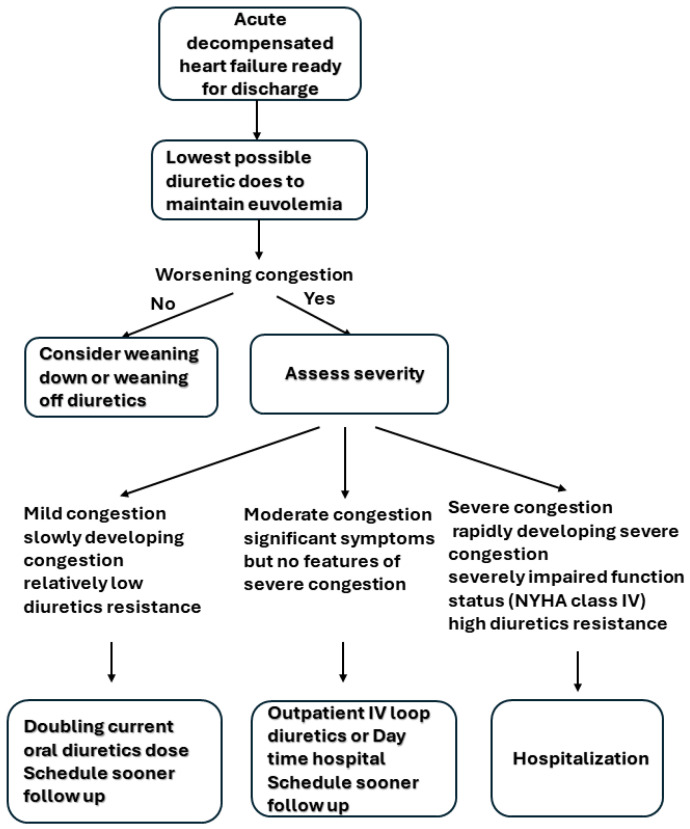
Algorithm stepwise approaches for how to use diuretics in outpatient HF.

On the other hand, the prompt recognition of heart failure and the triage of worsening chronic heart failure patients based on severity can prevent hospitalization. For moderately and slowly developing congestion with a relatively low diuretic resistance, an increase in oral diuretics (e.g., double the current dose) may be a reasonable first step. For patients with rapidly developing severe congestion, severely impaired function status (NYHA class IV), or high diuretic resistance, hospitalization is often needed [45]. For patients who fall in between the two sides of the spectrum, outpatient intravenous diuretics administration or day hospital has been proposed as an alternative to hospitalization in recent years [46,47]. A meta-analysis of 11 studies and 984 patients receiving outpatient intravenous loop diuretics showed that the re-hospitalization rates for HF at 30 and 180 days were 28 and 46%, respectively [48]. A protocolized outpatient management of diuretics among patients with worsening congestive heart failure has been proposed (Figure 2) [45].

## 6. Adjuncts to Diuretic Treatment

### 6.1. Thiazides and Thiazides-like Diuretics

Thiazide diuretics target the sodium-chloride cotransporter on the luminal side of the distal convoluted tubule [8]. When acting alone, thiazides exert a weak diuretic, as this segment of the renal tubule only accounts for 5–10% of the total sodium reabsorption [8]. In contrast, when combined with loop diuretics, thiazide blocks the compensation response of the distal convoluted tubule to loop diuretics, thereby achieving a “sequential nephron blockade” [20]. Such a combination can often overcome the loop diuretic resistance and produce robust diuresis. The two most used thiazides or thiazide-like diuretics in this setting are chlorothiazide and metolazone. Metolazone is only available as oral tablets, and chlorothiazide is primarily used as an intravenous regimen due to its lower bioavailability. The onset of action of intravenous chlorothiazide is about 30 min, whereas oral metolazone typically reaches the maximum plasma level in 1.5 h. In contrast, the half-life of metolazone is about 12–24 h, which is longer than chlorothiazide’s 6–12 h [49]. Although the combination of thiazide diuretics and loop diuretics is widely used in clinical settings and supported by observational studies, the data from RCT are limited [50]. The efficacy of metolazone (5 mg twice daily) and chlorothiazide (500 mg twice daily) has been evaluated and compared in a self-controlled RCT, named the 3T trial (*n* = 60), which demonstrated a similar efficacy in reducing weight among patients with heart failure and diuretic resistance [51]. More recently, the CLOROTIC trial evaluated the addition of hydrochlorothiazide (HCTZ) to loop diuretics (25–100 mg daily depending on renal function) and found that the combination was associated with a greater weight loss and urine output among patients with decompensated heart failure [52]. Compared to SGLT-2 inhibitors, thiazides in combination with loop diuretics were associated with the more frequent worsening renal function among patients with diuretic resistance, as shown in the DAPA-RESIST trial [24]. Electrolyte derangement, especially hypokalemia and hyponatremia, is common among patients who receive a combination of diuretic treatment, which requires frequent monitoring and the aggressive replacement of electrolytes [24,53].

### 6.2. Carbonic Anhydrase Inhibitors

Carbonic anhydrase (CA) inhibitors such as acetazolamide inhibit proximal convoluted tubule sodium bicarbonate reabsorption, thereby increasing sodium and bicarb excretion [54]. Loop diuretics alone promote renin releases and activate the neurohormonal response of the nephron by inhibiting the chloride uptake in the macula densa that promotes proximal tubular sodium bicarb absorption [11]. CA inhibitors, in combination with loop diuretics, are theorized to overcome diuretic resistance by blocking this compensatory response [55]. In the ADVOR trial, acetazolamide was associated with a higher decongestion rate (42.2 vs. 30.5%) and lower length of stay when combined with loop diuretics [56]. A subsequent analysis showed there was also a significant interaction between the effect of acetazolamide and the baseline renal function where the estimated glomerular filtration rate (eGFR) ≤40 mL/min/1.73 m^2^ was associated with a greater effect but also a greater risk of worsening renal function (defined as a creatinine rise of more than 0.3 mg/dL) [57]. Interestingly, a higher pretreatment serum HCO_3_ ≥ 27 mmol/L was associated with a greater effect of acetazolamide, whereas the development of a higher serum HCO_3_ was associated with worse clinical outcomes in the placebo group [58]. This finding is consistent with the theory that the compensatory activation of the neurohormone system during heart failure enhanced proximal nephron NaHCO_3_ reabsorption, which contributes to diuretic resistance.

### 6.3. Mineralocorticoid Receptor Antagonist

The mineralocorticoid receptor antagonist (MRA) inhibits the aldosterone-responsive epithelial Na channel (ENaC) in distal nephrons and collecting tubules, thereby limiting sodium absorption and potassium secretion [8]. Low-dose MRA has been proven to improve CV outcomes among patients with heart failure and a reduced ejection fraction in landmark trials without producing any significant natriuretic response [59]. Higher-dose (or diuretic-dose) MRA was theorized to synergistically work with loop diuretics by blocking RASS activation and producing more profound diuretics, similar to its use in cirrhosis [60]. However, in the ATHENA-HF trial, high doses (100 mg) of spironolactone did not result in a more significant reduction in N-terminal-pro B-type natriuretic peptide (NT-proBNP) or increase in urine output at 96 h when compared to no or low-dose (25 mg) spironolactone among patient with acute decompensated heart failure [61]. Although MRA may play a lesser role in overcoming diuretic resistance in the acute setting, it is still safe and beneficial to initiate MRA during acute decompensated heart failure for its neurohormonal effect and potassium-preserving effect [62]. MRA is contraindicated in patients with persistent hyperkalemia (K>5.5 mEq/L), and the evidence of the use of MRA among patients with heart failure and advanced staged chronic kidney disease (CKD) is limited.

### 6.4. Vasopressin Antagonists

Vasopressin antagonists act on V2 receptors in the distal nephron and collecting tubule, reducing free water reabsorption via the downregulation of the aquaporin (water) channel density [63]. This process is independent of the reabsorption of sodium and causes minimal neurohormone activation [64]. It has been observed that, during acute heart failure decompensation, there is an inappropriate elevation of arginine vasopressin, which contributes to fluid retention and hyponatremia [64]. It is, therefore, proposed that the addition of vasopressin antagonists can synergistically augment loop-diuretics-induced diuresis and improve hyponatremia among patients with acute congestive heart failure. In the EVEREST trial, tolvaptan was associated with an improvement in shorter-term heart failure symptoms but not all-cause mortality, composite CV mortality, or heart failure rehospitalization [65]. Notably, tolvaptan was effective in improving serum sodium levels among patients with hyponatremia (mean improvement in serum sodium level 5.49 vs. 1.85 mEq/L) [65]. Another pilot study compared tolvaptan against a standard dose of furosemide (40 mg) among patients hospitalized for acute congestive heart failure and found a similar efficacy of tolvaptan in achieving decongestion with a better preservation of renal function and less activation of the renin–angiotensin system compared to loop diuretics [66]. The use of tolvaptan should be limited to the short term as it has been associated with the incidence of liver dysfunction [67]. The role of a novel drug combining the V1a/V2 vasopressin antagonist (pecavaptan) in acute congestive heart failure is currently being investigated [68].

### 6.5. SGLT-2 Inhibitor

The sodium-glucose cotransporter-2 (SGLT-2) is located in the S1 and S2 segments of the proximal convoluted tubule, which is responsible for approximately 90% of the reabsorption of filtered plasma glucose [69]. The inhibition of SGLT-2 was traditionally believed to promote diuresis by osmotic diuretic decongestion mediated by glucosuria. However, this has not been supported by clinical observations [70]. More recently, SGLT-2 inhibitors were discovered to promote natriuresis by decreasing the Na+/H+ exchanger 3 (NH3) and bicarb reabsorption in the proximal tubule, which accounts for about 40% of filtered sodium [71]. Like CA inhibitors, SGLT-2 inhibitors do not lead to the compensatory activation of the renin–angiotensin–aldosterone system (RAAS). It helps mitigate the diuretic resistance by blocking the proximal convoluted tubule reabsorption of sodium bicarbonates when combined with loop diuretics. In the EMPAG-HF trial, the early (first day of hospitalization) introduction of empagliflozin among acute decompensated heart failure patients leads to an increased urine output and improved diuretics efficacy (urine per milligram furosemide) without negatively affecting the renal function [72]. In the EMPULSE trial, the initiation of empagliflozin during the acute decompensation of heart failure was associated with a significant clinical benefit (composite outcome of death, heart failure readmission, and symptom burden) as well as a greater weight reduction [73]. In the DICTATE-AHF trial, the early initiation of dapagliflozin, although it was not associated with a statistically significant reduction in weight-based diuretic efficiency (*p* = 0.06), it was associated with an improved median 24 h natriuresis (*p* = 0.03) and urine output (*p* = 0.005) in patients with acute congestive heart failure [74]. The effect of synergism SGLT-2 inhibitors, in addition to loop diuretics, appears less robust than those with thiazide-like diuretics such as metolazone. However, it is also associated with less fluctuation in electrolytes and renal function [24]. In addition, it should be noted that SGLT-2 inhibitors in recent years have established their central role in guideline-directed medical therapy (GMDT) among all heart failure patients, irrespective of the ejection fraction [2,3]. Its pleiotropic effect in improving diabetes, slowing the progression of CKD, and reducing the risk of atrial fibrillation should be considered, along with its diuretic effect when used among patients with heart failure [75,76]. The use of SGLT-2 inhibitors is limited in patients with advanced CKD with an eGFR < 20 mL/min/1.73 m^2^ due to the lack of evidence. The SGLT-2 inhibitors are also contraindicated in patients with type 1 diabetes due to the risk of diabetic ketoacidosis (DKA).

### 6.6. Inotropes

Inotropes are primarily used in the management of cardiogenic shock characterized by a low cardiac output and end-organ hypoperfusion. Diuretic agents are often ineffective alone in the state of cardiogenic shock due to poor perfusion to the kidney. Therefore, inotropes are helpful in restoring kidney perfusion and improving diuretic responsiveness. However, inotropes have not been shown to have mortality benefits in patients with severe heart failure but rather have been associated with increased adverse effects and mortality [77,78]. Among the inotropes, dopamine is a unique vasoactive agent as it exerts different physiological effects at different doses. At higher doses, dopamine has inotropic and vasoconstrictive effects, whereas, at lower doses (<3 μg/kg/min), it exerts selective vasodilation in the kidney [79]. It is then speculated whether low-dose dopamine can help optimize kidney function and improve diuretic effectiveness during decongestion. The ROES -AHF trial enrolled a total of 360 patients with decompensated heart failure and found that neither low-dose dopamine nor low-dose nesiritide (a vasodilator) was associated with more effective decongestion or improved renal function when added to diuretic therapy [80]. A post hoc analysis of ROSE AHF did show a better response to dopamine from patients with heart failure and reduced the ejection fraction compared to those with preserved ejection fraction, but the study sample size was not powered enough to make a conclusion [81]. In the OPTIME-CHF trial, routine use of short-term milrinone for acute decompensated heart failure also failed to shorten the hospital stay and was associated with an increased risk of arrhythmia [82]. Given the fact that the use of traditional inotropes has been associated with an increased risk of side effects and mortality, its use among patients with heart failure should be limited to patients with signs of a poor cardiac output with lowest dose and shortest duration if possible. Its main role in the contemporary management of heart failure is often as a bridge to more advanced therapy or heart transplant [83]. Digoxin has been proven to reduce HF hospitalization but not mortality among patients with heart failure and a reduced ejection fraction on GDMT [84]. Digoxin has a narrow therapeutic window and has been found to have frequent drug–drug interaction with various medications, including diuretics [85]. In a large observational study of 154,058 patients with heart failure, diuretics were associated with a three-fold increased risk of digoxin toxicity [86]. Newer inotropic agents, such as myotropes and cardiac contractility modulation, have emerged in recent years. Their roles in decongestion and acute decompensated heart failure remain to be seen in prospective clinical trials [87,88].

## 7. Monitoring and Adverse Effects of Diuretics

### 7.1. Hypokalemia and Hypomagnesemia

Loop diuretics increase the urinary excretion of potassium by blocking the sodium-potassium-chloride cotransporter-2 (NKCC2) and increasing the delivery of sodium to the collecting tube, which promotes the secretion of potassium and proton into the urine [89]. The latter mechanism is also shared by thiazide, and a combination of both types of diuretics can cause severe hypokalemia [89]. The blockage of the NKCC2 cotransporter at the thick ascending limb also diminishes the luminal positive transepithelial potential difference, which further reduces paracellular divalent cations reabsorption, including magnesium and calcium [90]. Hypokalemia and hypomagnesemia are very common among heart failure patients, especially those who received diuretics [91]. Hypokalemia and hypomagnesemia can cause various neuromuscular symptoms, including paresthesia, and weakness, as well as life-threatening arrythmia [92]. It is important to monitor the potassium and magnesium level closely during aggressive diuresis, and the potassium level should be promptly replaced to at least 3.5 mEq/L [93]. The early initiation of MRA and other RAAS inhibitors is also beneficial in maintaining the potassium level in heart failure patients [93].

### 7.2. Hyperkalemia

Hyperkalemia is a common side effect of MRA. The risk of hyperkalemia is higher among patients who have chronic kidney disease or a higher baseline potassium level or those who also take other RAAS inhibitors [94]. In the EMPHASIS trial, about 75% of patients experienced a potassium level of more than 4.5 mEq/L, and 9% of patients experienced a potassium level of more than 5.5 mEq/L, although life-threatening hyperkalemia is rare [95]. Currently, HF guidelines recommend against starting MRA among patients with a baseline potassium level of more than 5.0 mEq/L to avoid life-threatening hyperkalemia [2,3]. Potassium binders such as patiromer or sodium zirconium cyclosilicate can be used in this setting to improve the tolerability of MRA for heart failure patients [96].

### 7.3. Hyponatremia

Hyponatremia is frequently observed among patients with heart failure and is associated with a worse prognosis [97]. The mechanisms of hypotonic hyponatremia included dilutional and depletional hyponatremia [98]. An increased vasopressin release due to neurohormone activation and a decreased vasopressin degradation due to liver and kidney dysfunction contributed to the free water retention and thirsty feeling [64]. The reduced distal nephron flow due to the enhanced proximal solute/water reabsorption limits the dilution process of the urine [99]. On the other hand, the low solute intake, hypokalemia/hypomagnesemia, and excessive natriuresis from diuretics, especially the combination of loop diuretics and distal nephron-acting diuretics, can also lead to sodium depletion and cause hyponatremia [98]. A clinical assessment and volume status examination can help differentiate dilutional and depletional hyponatremia in patients with heart failure receiving diuretics. Urine osmolarity of less than 100 mOSm/L and urine sodium of less than 50 mEq/L usually suggest depletional hyponatremia [98]. Stopping the distally working diuretics such as thiazide and MRA and repleteing potassium/magnesium will help both types of hyponatremias. For dilutional hyponatremia, limiting the free water intake is generally the first step, and vasopressin antagonists can be considered in severe cases. For depletional hyponatremia, the scaling back of diuretics should be considered, and an intravenous hypertonic saline infusion can be considered in severe cases with special attention to the rate of correction to avoid central pontine myelinolysis [100].

### 7.4. Worsening Renal Function

Worsening renal function (WRF), commonly defined as a higher than 0.3–0.5 mg/dL rise in creatinine (Cr) or a 20–30% rise in glomerular filtration rate (GFR) compared to baseline, has been frequently observed during acute decompensated congestive heart failure and has been frequently observed in patients with acute congestive heart failure receiving diuretics [101,102]. WRF has been associated with poor outcomes among patients with decompensated heart failure in observational studies [103,104]. Recently, it has been increasingly recognized that not all forms of WRF are associated with worse clinical outcomes. In the case of aggressive diuresis or the initiation of guideline-directed medical therapy, WRF seems to have minimal impact on the prognosis. In the ESCAPE trial, among 433 enrolled patients with acute congestive heart failure and reduced ejection fraction, 36% of patients developed WRF (defined as a rise in Cr of more than 0.3 mg/dL). Among patients who were considered decongested at discharge, in-hospital WRF was not significantly associated with 180-day all-cause death [105]. In contrast, those who developed WRF and remained congested upon discharge had a significantly higher risk of 180-day rehospitalization and all-cause mortality compared to those without [105]. Similarly, in the DOSE trial, a higher dose of loop diuretics was associated with a higher risk of WRF (defined as a rise in Cr of more than 0.3 mg/dL), but WRF was not associated with a worse outcome [106]. The mechanism of the loop-diuretics-induced drop in GFR is through the increased macula-densa-mediated renin release and the blockage of the tubuloglomerular feedback mechanism rather than a true tubular injury [107]. In the ROSE-AHF trial, there is no correlation between changes in the serum creatine and changes in the makers of tubular injury such as Kidney Injury Molecule-1 (KIM-1), Neutrophil gelatinase-associated lipocalin (NGAL), and N-acetyl-β-d-glucosaminidase (NAG) among patients who were treated with aggressive diuresis [108]. A rise in creatinine during acute decongestion is often transient if successful decongestion is achieved [23,105]. Given the above evidence, it is proposed that such a transient drop in GFR during decongestion or up-titration of GDMT be classified as “pseudo-WRF” [109]. In contrast, a gradual and unprovoked decrease in GFR among patients with chronic congestive heart failure (Type 2 cardiorenal syndrome) is associated with poor clinical outcomes, as it reflects nephron loss due to intraglomerular hypertension or chronic hypoperfusion damage [109,110]. In addition, acute kidney injury presented on HF admission (Type 1 cardiorenal syndrome) followed by worsening renal function is often associated with worse clinical outcomes [111]. Therefore, the monitoring of kidney function during and after acute heart failure hospitalization is important and may provide prognostic information. In summary, a modest rise in creatine among congested patients receiving aggressive diuretics therapy is acceptable so long as the patient is responding well to the diuretics. The response to diuretics should be measured systemically, as stated above, so that patients with a poor response can be identified [109]. In the face of significantly worsened renal function, alternative causes of acute kidney injury, such as hypotension, obstruction, and nephrotoxin, should be sorted.

### 7.5. Hyperuricemia and Gout Attack

Both loop diuretics and thiazides promote urate reabsorption by the proximal tubule, which causes hyperuricemia and predisposes patients with heart failure to develop gout [112]. Loop diuretics, compared to thiazides, are associated with a higher risk of gout [113]. It estimated that a gout attack occurs in 3.6% of acute decompensated heart failure [114]. The development of gout is not necessarily an indication of stopping the diuretics. It can be managed by urate-lowering agent such as allopurinol in combination with acute gout flare medication such as colchicine or prednisone [115]. NSAID is contraindicated in the management of gout among heart failure patients [115].

### 7.6. Hypersensitivity

Many diuretics, including furosemide, bumetanide, and torsemide, are all sulfonamides, which may cause hypersensitivity reactions such as skin rashes or rarely acute interstitial nephritis [116]. Ethacrynic acid, which is a non-sulfonamide loop diuretic, can be used instead but it is associated with a higher risk of ototoxicity [117]. There appears to be minimal cross-reactivity of hypersensitivity to antimicrobial sulfonamides such as sulfamethoxazole, versus non-antimicrobial sulfonamides such as loop diuretics [118]. Therefore, patients who are allergic to antimicrobial sulfonamides are not necessarily contraindication to loop diuretics. Detailed history-taking and consultation with pharmacists are recommended.

### 7.7. Ototoxicity

Loop diuretics can cause ototoxicity, which can present as reversible hearing loss or tinnitus, and rarely permanent deafness [119]. The ototoxicity of loop diuretics often occurs with high doses and rapid intravenous infusion (furosemide dose above 240 mg/h or 4 mg/min) but can also occur at lower doses in patients with acute kidney injury and patients taking other ototoxins such as aminoglycoside [120,121]. Continuous infusion, compared to bolus, appears to have a lower risk of ototoxicity due to the lower peak serum concentration [122]. A dose higher than 80 mg of furosemide is best given via infusion rather than bolus dosing. Among loop diuretics, ethacrynic acid is associated with a higher risk of ototoxicity compared to the rest of the loop diuretics [117].

## 8. Diuretics in Certain Phenotypes

### 8.1. Diuretics in Chronic Kidney Disease/End-Stage Renal Disease

Chronic kidney disease (CKD) and heart failure frequently co-exist and are associated with worse heart failure outcomes [104,123]. Diuretics use in CKD patients is often challenging due to the diminished response to diuretics. The secretion of diuretics to the tubular lumen is impaired in patients with CKD due to a decline in GFR and loss of function in nephron [124]. Therefore, the ceiling dose of intravenous diuretics is usually higher for patients with CKD, which can be as high as 160–200 mg, and the daily total dose can be as high as 600 mg of furosemide or an equivalent dose of bumetanide or torsemide [125]. Distal nephron sodium delivery is increased in patients with CKD due to reduced proximal sodium reabsorption. Thus, thiazide is also an ideal diuretic in CKD [126]. Thiazide can be used in combination with loop diuretics or alone in CKD patients and has been shown to effectively reduce fluid retention and improve blood pressure [127]. A significant portion of end-stage renal disease (ESRD) patients on dialysis have residual kidney function and continue to make urine [128]. Residual kidney function has been associated with improved survival in ESRD patients [129]. Loop diuretics have been shown to be beneficial in maintaining the fluid balance among patients with ESRD [130]. Due to the significantly diminished GFR, the effective dose of loop diuretics among patients with ESRD is often much higher than those without ESRD and are frequently combined with thiazide to achieve efficacy [131].

### 8.2. Diuretics in Pregnancy

Congestive heart failure affects about 1 in 1000 to 1 in 10,000 pregnant women and is associated with an increased risk of adverse maternal–fetal outcomes [132,133]. Per clinical guidelines, the use of diuretics is recommended for the symptom relief of congestive heart failure and volume optimization during pregnancy [134,135]. There is concern that the use of diuretics may cause a drop in placental blood perfusion and adverse fetal outcome [136]. This has not been supported by a meta-analysis of 20 randomized trials that showed no increased risk of stillbirth with the use of furosemide or thiazides [137]. The use of diuretics should be individualized, and caution should be taken to avoid volume depletion. Among loop diuretics, the use of furosemide has been well-documented in the literature, whereas the data for torsemide and bumetanide are limited. Loop diuretics can also suppress lactation and transfer to the infant via breast milk; therefore, infant follow-up is necessary [136]. Spironolactone should be generally avoided during pregnancy due to its hormonal effect.

### 8.3. Diuretics in Elderly

The use of diuretics in elderly patients with heart failure is challenging due to the increased risk of side effects. Some of these side effects are due to intrinsic physiologic changes due to aging, such as the decline in GFR and body mass, and the unpredictability of drug absorption [138,139]. More commonly, the use of diuretics can be interfered with by the coexisting cognitive and mobility dysfunction and drug interaction from polypharmacy. For example, elderly patients are prone to hypovolemia from diuretics due to decreased total body water, a decrease in maximal urinary concentrating capacity [140], and, oftentimes, the decreased ability to maintain hydration [141]. This is commonly associated with orthostatic hypotension and falls [142]. A meta-analysis of 62,721 elderly patients has shown the probability of falls in the elderly who used diuretics was 1.2 times higher than that in the elderly who did not use diuretics [143]. NSAIDs have been found to be associated with a two-fold increased risk of hospitalization for CHF among elderly patients taking diuretics [144]. These real-world studies highlight the need for the frequent monitoring of diuretics use in the elderly and scrutinization of the medication that is being co-administrated.

### 8.4. Diuretics in Heart Failure with Preserved Ejection Fraction (HFpEF)

Diuretics are the cornerstone of managing patients with heart failure and preserved ejection (HFpEF). HFpEF is a heterogenic disease that is often linked to underlying etiologies [145]. Until recently, diuretics were the only GMDT that is universally recommended for all patients with HFpEF, in addition to the management of the underlying etiology or comorbidities. With the arrival of newer clinical trials such as EMPEROR-Preserved, DELIVER, and TOPCAT, loop diuretics adjuncts such as SGLT-2 inhibitors and MRA have also been recognized for their role in improving HF hospitalization for patients with HFpEF [146,147,148]. HFpEF patients may respond to loop diuretics differently compared to heart failure with reduced ejection fraction (HFrEF) patients during acute decompensation. A secondary analysis of the DOES trial suggested that aggressive diuresis during acute heart failure was associated with an elevated risk of worsening renal function without significantly improved fluid removal compared to HFrEF patients [149]. This is thought to be related to the fact that HFpEF tends to have more interstitial fluid retention rather than intravascular volume expansion when compared to HFrEF [150]. This finding needs to be validated in future clinical trials.

### 8.5. Diuretics in Right Ventricular Failure

Right ventricular (RV) failure is defined as heart failure symptoms as a result of RV dysfunction. RV dysfunction is common among patients with congestive heart failure. In a meta-analysis of HFrEF patients, the prevalence of RV failure was 48% [151]. Common causes of RV dysfunction include ischemic cardiomyopathy, precapillary or postcapillary pulmonary hypertension, atrial fibrillation, obesity, and left ventricular assistive device [152]. The presence of RV dysfunction is an independent predictor of short- and long-term mortality in patients with heart failure [153]. The volume management of RV failure faces a unique dilemma. While some patients with RV failure are preload-dependent, the majority of patients with RV failure will have an elevated RV filling pressure and central venous pressure. A judicious volume repletion is indicated in those with a low central venous pressure and low arterial blood pressure, whereas diuretics should be given to patients with significant venous congestion [154]. Invasive hemodynamic monitoring is often helpful to guide volume management in such patients [154]. RV failure often leads to cardiorenal syndrome characterized by the venous congestion of kidneys and a decrease in GFR. This, in return, worsens the diuretic resistance, thereby creating a vicious cycle. The early identification of diuretic resistance and combination therapy is crucial in achieving decongestion for RV failure patients [155].

### 8.6. Diuretics in Heart Transplant

Heart transplant remains the gold standard treatment for patients with advanced heart failure [156]. The high-dose diuretics requirement has been linked to a significantly higher risk of waitlist death among ambulatory patients waiting for a heart transplant, likely as an indicator of residual congestion [157]. Hypertension and fluid retention are frequently observed among post heart transplant patients [158]. This has been postulated to be due to a lack of reflex-mediated suppression of fluid regulatory hormones [159]. Calcineurin inhibitors such as cyclosporine and tacrolimus are known to cause renal magnesium waste [160]. The use of diuretics among heart transplant patients who are on calcineurin inhibitors can further lead to electrolyte derangement. Loop diuretics were found to have less impact on the magnesium level compared to thiazide diuretics among patients on calcineurin inhibitors [161].

## 9. Current Challenges

### 9.1. Diuretic Resistance

Diuretic resistance represents a major challenge of diuretic therapy during congestive heart failure [6]. Diuretic resistance is often defined as the failure to decongest despite adequate and escalating doses of diuretics [162]. Diuretic resistance is not uncommon in clinical practice. In a study by the Spanish Heart Failure Registry (RICA), diuretic resistance, which is defined by heart failure rehospitalization despite receiving more than 80 mg of furosemide per day, was found to be in more than 21% of admitted patients with congestive heart failure [163]. Diuretic resistance is associated with major clinical adverse outcomes. From the same study by the RICA registry, diuretic resistance is associated with a significantly increased one-year mortality among heart failure patients with a hazard ratio of 1.37 [163]. Risk factors of diuretic resistance include elevated serum creatinine, a lower systolic blood pressure, lower serum chloride, older age, and female sex [164].

There are many proposed mechanisms of diuretic resistance, which can be divided into prerenal and intrarenal causes (Table 2). The prerenal cause of diuretic resistance includes venous congestion causing gut edema that leads to the decreased absorption of diuretics [15]; a low cardiac output and/or increased abdominal pressure that decreases renal blood flow and GFR [165]; and hypoalbuminemia that promotes fluid third spacing and a decline in effective intravascular volume, and reduces the active uptake of loop diuretics from the nephron [166]. The intrarenal cause is often related to the nephron loss in the setting of acute or chronic kidney injury that reduces the tubular secretion of diuretics. The neurohormonal activation leads to compensatory enhancement of sodium/water reabsorption at the distal convoluted tubule and collecting tubule [13]. Loop diuretics also directly inhibit the chloride uptake in the macula densa by blocking the NKCC receptor, which activates the renin release and its downstream angiotensin and aldosterone upregulation [10]. As a result, the proximal nephron sodium and bicarbonate reabsorption and distal sodium/water reabsorption are also enchanted, which offset the effect of loop diuretics [11]. Hypochloremia alkalosis has been traditionally due to volume depletion (contraction alkalosis) and is often suggested to be an indication of over-diuresis [167]. This has been challenged by the recent observation that both hypochloremia and hyperbicarbonatemia may also represent a result of neurohormone activation during decompensated heart failure rather than volume depletion [168,169]. Diuretic resistance should be differentiated from diuretics’ “braking phenomenon”, which is a normal physiological response to the repetitive dosing of loop diuretics to avoid a drastic volume depletion [20].

The strategy of overcoming diuretic resistance is rapidly evolving. With the arrival of studies such as the ADVOR, EMPULSE, and CLOROTIC trials, more and more options have become available to address diuretic resistance. Before adding more diuretics adjuncts, diuretics dosing should be optimized, and external factors such as increased abdominal pressure and urinary obstruction should be addressed [4]. Thiazide and thiazide-like diuretics are widely used clinically for the sequential nephron blockade strategy and have been recommended by the clinical guidelines [2,3]. However, evidence of their use is still scarce from prospective clinic trials, and it has been associated with significant electrolyte derangement and an increased risk of worsening renal function [24,53]. Carbonic anhydrase inhibitor carries the advantages of a neutral effect on neurohormonal activation and appears to be associated with less electrolyte derangement and kidney dysfunction [56]. It is particularly useful among patients with hyperbicarbonatemia. Vasopressin antagonists also have a relatively neutral effect on neurohormone activation and should be considered among patients with severe hyponatremia. The SGLT-2 inhibitors have been proven to be beneficial and safe when added to loop diuretics, although it appears less effective in achieving durable diuresis when compared to thiazides [24,170]. Importantly, the SGLT-2 inhibitors are associated with improved clinical outcomes for acute congestive heart failure patients and play an important role in the long-term management of heart failure [73]. The early recognition of patients at risk or in the early phase of cardiogenic shock and timely use of therapy such as inotropes and more advanced therapy are crucial in achieving decongestion and preserving renal function in these high-risk patients [171].

### 9.2. Incomplete Decongestion

Another major challenge to diuretics used in acute congestive heart failure is incomplete decongestion before discharge. It is estimated that about one-third of heart failure patients were discharged with residual congestion, which is associated with a significant risk for HF readmission and mortality [172,173]. This is often multifactorial due to the administrative pressure upon clinicians to discharge sooner, and the failure to recognize residual congestion, as well as an intrinsic advanced state of congestive heart failure that resulted in resistance to diuretics [174].

In recent clinical trials, it has been increasingly recognized that residual congestion is an important clinical outcome, and its measurement can be standardized. In the DOSE trial, freedom from congestion is defined as jugular venous pressure (JVP) < 8 cm H_2_O, no more than trace peripheral edema, and an absence of orthopnea, and this only occurs in 11–18% of patients in different treatment groups at 72 h after admission [23]. In comparison, the ADVOR trial used a congestion score on a scale of 0–10 (peripheral edema, pleural effusion, and ascites). Decongestion was achieved in 42.2% of the acetazolamide group and 30.5% of the placebo group [56]. These approaches remain to be tested in future prospective studies. Biomarkers such as BNP, hemoconcentration markers such as hemoglobin/hematocrit, and protein/albumin have been proposed to be surrogates for decongestion and have been shown to correlate with clinical outcomes [175,176,177]. However, these marks are often subjected to other confounding processes; therefore, it is best to combine them with clinical assessment [178]. Invasive hemodynamic measurement and target therapy, in addition to the standard clinical assessment, have not been proven to affect heart failure hospitalizations or mortality in ESCAPE trials [179]. But, more recently, an implantable pulmonary artery pressure measurement device has been suggested to effectively reduce heart failure hospitalization in GUIDE-HF trials [180].

As stated above, a moderate elevation in creatinine is common during the decongestion or titration of GDMT is often not associated with adverse long-term cardiovascular (CV) outcomes. Therefore, it should not be the sole reason to hold back diuretics. It is, however, important to reassess the hemodynamic profile and the other contributing factors that could affect the kidney function in such patients and consider adjusting the diuretic strategy if necessary.

## 10. Future Direction

Diuretics have been the cornerstone for the management of heart failure for decades and have undoubtedly improved the outcome of patients with heart failure. In the era of evidence-based medicine, further studies are needed to better delineate the optimized diuretic strategy for acute decompensated heart failure, diuretic resistance, and chronic congestive heart failure. The standardized and protocol-driving diuretics strategy and measurement of decongestion, as shown in recent clinical studies, have been proven to provide more consistent outcomes and allow for a comparison of clinical outcomes between clinical trials. Its impact on survival benefits and the long-term outcome of heart failure is still unclear. The early identification of patients at risk for diuretic resistance and the involvement of multidisciplinary care is crucial in patients with high-risk heart failure. With more and more evidence emerging for newer adjuncts to traditional diuretics, it is also important to study the long-term CV and renal outcomes of these agents. An optimized strategy of diuretics remains to be defined for ambulatory diuretics use in patients with chronic heart failure. It might be beneficial to down-titrate or withdraw diuretics in patients with improved cardiac function after medical therapy. Novel approaches such as implantable hemodynamics monitoring provide a promising future for hemodynamic-guided diuretics treatment.

## Figures and Tables

**Table 1:** Summary of clinical trials or landmark studies for diuretics.

Trials	Intervention/Medication	Patient Population	Outcome Measured	Conclusions
DOES	(2 × 2 factorial design) Intravenous bolus versus continuous infusion of furosemide; Low dose (1× oral dose) versus high dose (2.5× oral dose) of furosemide.	Acute decompensated heart failure; N = 308; Mean ejection fraction (EF) = 35%.	Primary outcome: Global symptom relief by visual analog scale (VAS) area under the curve (AUC); renal function; Secondary outcome: Change in weight; net fluid loss; decongestion; death, rehospitalization, emergency room visit.	No difference in symptom relief or changes in creatine between intravenous bolus versus continuous infusion or low dose versus high dose of furosemide; High-dose furosemide was associated with greater net fluid loss, weight loss and improvement in dyspnea compared to low-dose furosemide.
CARRESS-HF	Slow continuous ultrafiltration versus stepped pharmacological treatment of loop diuretics.	Acute decompensated heart failure (ADHF) with worsened renal function (increase of Cr more than 0.3 mg/dL), with persistent congestion despite intravenous diuresis; N = 188; Median EF = 33%.	Primary outcome: Change in serum creatinine (Cr) and change in weight between randomization and 96 h; Secondary outcome: Rate of clinical decongestion, global well-being, and dyspnea	Slow continuous ultrafiltration was associated with a worsening primary endpoint (driven by worsening serum Cr) compared to stepped pharmacological treatment; Similar rate of weight loss and decongestion.
ENACT-HF	Protocolized natriuresis-based diuretics dosing versus standard of care.	Acute decompensated heart failure; N = 401; EF: 55% of patients had EF < 40%.	Primary outcome: Natriuresis after 24 h; Secondary outcome: Diuresis, weight loss, change in congestion score and length of stay.	Protocol-driven and natriuresis-based diuresis was associated with higher natriuresis, higher urine output, and shorter length of stay; There was no difference in motility between the two arms.
Transform HF	Torsemide versus furosemide.	Discharged patient with decompensated heart failure; N = 2859; EF: 64% of patients had EF <= 40%.	Primary outcome: All-cause mortality; Secondary outcome: All-cause mortality or hospitalization; quality of life.	No difference in all-cause mortality in one year; No difference between groups for the composite of all-cause mortality or all-cause hospitalization at 12 months; No difference in quality of life between the two groups.
3T Trial	Oral metolazone, intravenous chlorothiazide, or tolvaptan therapy in addition to loop diuretics.	Acute decompensated heart failure with diuretic resistance (furosemide equivalent dosage of >=240 mg/day with persistent congestion); N = 60; Mean EF = 30%.	Primary outcome: 48 h weight loss; Secondary outcome: 48 h total and net urine output; congestion symptoms; diuretic efficiency.	Among patients with loop diuretic resistance, the addition of oral metolazone, intravenous chlorothiazide, or tolvaptan therapy all resulted in significant weight reduction; There was no between-group difference in the primary outcome.
CLOROTIC	Hydrochlorothiazide versus placebo, in addition to intravenous loop diuretics.	Acute decompensated heart failure; N = 230; Mean EF = 56%.	Primary outcome: Changes in body weight and patient-reported dyspnea 72 h after randomization; Secondary outcome: Diuretic response; mortality/rehospitalizations at 30 and 90 days.	Hydrochlorothiazide, in addition to intravenous loop diuretics, was associated with greater weight loss at 72 h; There was no difference in relief of dyspnea between the two groups; Hydrochlorothiazide was associated with improved diuretic efficiency but more frequent worsening of renal function.
EMPAG-HF	Early initiation (day one of hospitalization) of empagliflozin versus placebo in addition to standard medical treatment of acute decompensated heart failure.	Acute decompensated heart failure; N = 60; Mean EF = 45%.	Primary outcome: Total urine output measured and summed over 5 days; Secondary outcome: Renal function.	Use of empagliflozin 25 mg within 12 h of hospitalized ADHF patients was associated with a 25% increase in cumulative urine output in 5 days; Empagliflozin use was associated with a larger decrease in N-terminal-pro B-type natriuretic peptide (NT-proBNP) levels and improved NYHA class with a better eGFR at day 30 compared to placebo.
EMPULSE	Empagliflozin versus placebo, in addition to standard medical treatment of acute decompensated heart failure.	Acute decompensated heart failure; N = 530; Median EF = 32%.	Primary outcome: Composite of death, number of heart failure events, time to first heart failure event, and change in Kansas City Cardiomyopathy Questionnaire-Total Symptom Score (KCCQ-TSS) from baseline to 90 days; Secondary outcome: Acute renal failure; body weight change.	Empagliflozin was associated with significant clinical benefits of primary outcome (time to death, heart failure events, improvement of total symptoms score) after 90 days of treatment; Empagliflozin was also associated with greater weight loss changes in congestion score compared to placebo.
DAPA-RESIST	Dapagliflozin versus metolazone for heart failure with diuretic resistance.	Acute decompensated heart failure with diuretic resistance (receiving ≥160 mg IV furosemide in 24 h with insufficient decongestion); N = 61; Median EF = 45%.	Primary outcome: Diuretic effect (weight changes) from randomization to 96 h; Secondary outcome: Change in congestion; loop diuretic efficiency.	There was no difference in weight changes at 96 h between dapagliflozin and metolazone; Dapagliflozin groups had higher cumulative loop diuretic dosing requirements but were associated with less hypokalemia, hypotension, and worsening creatines.
DICTATE-AHF	Early initiation of dapagliflozin versus placebo in addition to standard medical treatment of acute decompensated heart failure.	Acute decompensated heart failure; N = 240; EF: 52% had EF <= 40%.	Primary outcome: Diuretic efficiency defined as cumulative weight change per cumulative loop diuretic dose; Secondary outcome: 24 h natriuresis and urine output.	Dapagliflozin was not associated with a statistically significant reduction in weight-based diuretic efficiency (*p* = 0.06); Dapagliflozin was associated with increased natriuresis (*p* = 0.03) and urine output (*p* = 0.005) at 24 h; Early initiation of Dapagliflozin was not associated with an increase in any safety events.
ADVOR	Intravenous administration of acetazolamide (500 mg daily) versus placebo in addition to standardized intravenous loop diuretics.	Acute decompensated heart failure; N = 519; Mean EF = 43%; Patient on SGLT-2 inhibitors were excluded.	Primary outcome: Successful decongestion within 72 h; Secondary outcome: composite end point of death from any cause or rehospitalization for heart failure during three months of follow-up; length of stay.	Acetazolamide was associated with a higher incidence of successful decongestion at 72 h; There was no difference in all-cause mortality and heart failure; Acetazolamide was associated with shorter length of stay.
ATHENA-HF	High-dose spironolactone versus low-dose or placebo for acute decompensated heart failure patient.	Acute decompensated heart failure; N= 360; Median EF = 34%.	Primary outcome: change in NT-proBNP levels; Secondary outcome: clinical congestion score; dyspnea assessment; net urine output; and net weight change.	High-dose spironolactone in ADHF was not associated with improvement in NT proBNP levels; There was also no difference in secondary outcome, including clinical congestion score, net urine out, or net weight change.
ROSE HF	Low-dose dopamine (2 μg/kg/min) or low-dose nesiritide (0.005 μg/kg/min without bolus) in addition to standard diuresis.	Acute decompensated heart failure with renal dysfunction; N = 360; Median EF = 33%.	Primary outcome: 72 h cumulative urine volume (decongestion endpoint); change in serum cystatin C from enrollment to 72 h (renal endpoint); Secondary outcome: change in creatine; dyspnea assessment, changes in weight, worsening heart failure, death, heart failure rehospitalization.	There was no difference in cumulative urine volume or changes in serum cystatin outcome between low-dose dopamine, low-dose nesiritide, and placebo; There was no difference in death HF hospitalization at 60 days between groups; Patients with ejection fraction ≤50% appeared to derive more benefit from dopamine (*p* for interaction = 0.01).
OPTIME-CHF	Short-term use of milrinone versus placebo in addition to standard therapy.	Acute decompensated heart failure not requiring inotropes supports; N = 951; Mean EF = 23%.	Primary outcome: Cumulative days of hospitalization within 60 days after randomization; Secondary outcome: Treatment failure caused by progression heart failure or adverse effect; hypotension; arrythmia.	The primary endpoint of cardiovascular hospital days at 60 days was not significantly different between milrinone and placebo groups; Sustained hypotension and new atrial fibrillation or flutter were more common for milrinone than placebo subjects.
CHAMPION	Implantable pulmonary artery pressure monitors to guide the medical management of patients with heart failure.	NYHA class III heart failure symptoms and recent hospitalization for heart failure; N = 2859; EF: 83% of patients had EF <= 40%.	Primary outcome: Rate of heart failure-related hospitalizations at six months; Secondary outcome: Change from baseline in pulmonary artery mean pressure, Minnesota Living with Heart Failure Questionnaire at six months; device complication.	The use of implantable pulmonary artery pressure monitors was associated with a significant reduction in HF hospitalization at six months; The effect of implantable pulmonary artery pressure monitors was durable at 18 months; Overall freedom from device-related complications was 98.6%.

**Table 2:** Causes of diuretic resistance and management.

Cause of Diuretic Resistance	Mechanism	Strategy to Overcome Diuretic Resistance
Prerenal cause		
Venous congestion, intestinal edema	Reduced absorption of loop diuretics from the gastrointestinal tract; Venous congestion causes increases in renal venous pressure, leading to a drop in glomerular filtration rate.	Use IV diuretics or diuretics that have better bioavailability.
Increased abdominal pressure	A rise in intrabdominal pressure causes increases in renal venous pressure, leading to a drop in Glomerular filtration rate.	Address constipation, urinary retention, and significant ascites.
Low cardiac output	Low cardiac output decreases kidney.	Inotropes, vasodilators, advanced therapy such as left ventricular assist device.
Hypoalbuminemia	Loop diuretics are highly protein-bound. Hypoalbuminemia reduces the level of active form of loop diuretics.	Albumin infusion (poor effect) improves nutrition.
Intrarenal cause		
Proximal tubular injury	Reduced delivery of loop diuretics to target site.	Treat underlying etiology of acute tubular injury.
Neurohormonal activation	Extracellular fluid volume contraction and activation of the renin–angiotensin–aldosterone system promotes the proximal solute reabsorption and decrease the delivery of sodium and chloride to the distal tubule.	Carbonic anhydrase inhibitor. Sodium-glucose cotransporter-2 inhibitors. Mineralocorticoid receptor antagonists and other guideline-directed medical therapy.
Compensatory reabsorption in distal convoluted tubule or collecting tubule	Epithelial cell of distal tubule undergoes both hypertrophy and hyperplasia in response to loop diuretics that increases solute delivery to distal segments.	Thiazide diuretics; Mineralocorticoid receptor antagonists
Free water excretion impairment	Excessive vasopressin leads to significant water retention and severe hyponatremia.	Vasopressin antagonist.
Albuminuria	Furosemide can bind to albumin within the tubular lumen, which reduces the level of active and unbound drug that is capable of binding to the tubular receptor.	Angiotensin-converting enzyme inhibitors and angiotensin receptor blockers, mineralocorticoid receptor antagonists.
